# Selective serotonin reuptake inhibitors (SSRIs) in women of reproductive age: a systematic review of local formularies

**DOI:** 10.3399/BJGPO.2023.0255

**Published:** 2024-05-29

**Authors:** Elizabeth Lovegrove, Alice Maidwell-Smith, Beth Stuart, Miriam Santer

**Affiliations:** 1 Primary Care Research Centre, University of Southampton, Southampton, UK; 2 Hampshire Hospitals NHS Trust, Southampton, UK; 3 Wolfson Institute of Population Health, Queen Mary University of London, London, UK

**Keywords:** anxiety, breast feeding, depression, maternal health, obstetrics, postpartum period, pregnancy, selective serotonin reuptake inhibitors, general practitioners, primary healthcare

## Abstract

**Background:**

Depression is the second most common chronic condition affecting women of reproductive age; 23.4% of women enter pregnancy with depression and use of selective serotonin reuptake inhibitors (SSRIs) in pregnancy is often necessary for maternal wellbeing. However, SSRI use during pregnancy can cause congenital malformations, postpartum haemorrhage (PPH), and persistent pulmonary hypertension of the newborn (PPHN). In UK primary care, prescribing formularies are one medium by which prescribers are provided with local medicines advice.

**Aim:**

To review all local prescribing formularies with respect to prescribing SSRIs in women of reproductive age, during pregnancy, and during breastfeeding.

**Design & setting:**

A systematic review of prescribing formularies in England and Wales.

**Method:**

A systematic keyword search of all clinical commissioning group and Integrated Care Board websites in England and Local Health Board websites in Wales was undertaken between December 2021–22 to identify prescribing formularies. Data were extracted on prescribing guidance for SSRIs.

**Results:**

Seventy-four prescribing formularies were reviewed. Of these, 14.9% (*n* = 11/74) provided links to the Medicines and Healthcare products Regulatory Agency guidance on congenital abnormalities associated with SSRIs, 28.4% (*n* = 21/74) provided links to guidance on PPH risk, and 1.4% (*n* = 1/74) provided links to guidance on PPHN. Specific local guidance was given on SSRI prescribing for women of reproductive age, during pregnancy, and during breastfeeding in 12.2% (*n* = 9/74), 23.0% (*n* = 17/74), and 21.6% (*n* = 16/74) of formularies, respectively.

**Conclusion:**

Our results suggest that prescribers may be poorly informed by local formularies about the risks of SSRI use around pregnancy. This could place babies at increased risk of unintentional SSRI exposure.

## How this fits in

Continuation of selective serotonin reuptake inhibitors (SSRIs) during pregnancy and postpartum is often essential to adequately treat maternal depression and anxiety. However, SSRI use during pregnancy carries small but significant risks to mother and baby. Women of reproductive age prescribed SSRIs should therefore be informed about these risks, ideally before conception so they can make informed decisions about future treatment and pregnancy plans. This study demonstrates that advice given to primary care prescribers regarding these risks is suboptimal, and may place women and babies at risk of unintended SSRI exposure during pregnancy.

## Introduction

National and international prevalence rate estimates for antidepressant use in women of reproductive age range from 11% to 20%.^1–4^ Depression is the second most common chronic condition affecting women of reproductive age, and depression and anxiety are the two most prevalent health conditions affecting pregnant women in the UK at 23.4% and 19%, respectively.^5,6^ Selective serotonin reuptake inhibitors (SSRIs), the medication class recommended for first line pharmacological management of depression and anxiety, are used in nearly 5% of all pregnancies and in 17.4% of pregnancies carried by women with two or more long-term conditions.^5–8^ SSRI use around pregnancy is likely to continue increasing alongside the prevalence of depression and anxiety; antidepressant prescription rates have doubled in the past decade, and prevalence of SSRI use for anxiety increased from 16.6/1000 person-years-at-risk (PYAR) to 34.9/1000 PYAR between 2003 and 2018.^9–15^ This trend is likely to continue to disproportionately affect women living in lower income households or more deprived areas; 17% of England’s poorest women receive antidepressants, versus 7% of the richest, and a similar pattern is seen by area deprivation.^2,3,13,16,17^

Untreated maternal mental illness can lead to increased risk of maternal pregnancy complications, preterm birth and low birth weight, postpartum suicidality, and offspring cognitive and behavioural difficulties.^18–21^ Indeed, mental illness was the fourth most common cause of maternal deaths in the UK between 2019–2020, and maternal suicide is the leading cause after 6 weeks postpartum.^22,23^ It is essential, therefore, for maternal and infant wellbeing that maternal mental health conditions are adequately treated and this may involve SSRIs.

However, the Medicines and Healthcare products Regulatory Agency (MHRA) 2014 alert highlighted an increased risk of congenital malformations when the SSRIs paroxetine and fluoxetine are used in the first trimester of pregnancy, and persistent pulmonary hypertension in the newborn (PPHN) when SSRIs are used close to delivery.^24,25^ A 2021 alert advised of the increased risk of postpartum haemorrhage (PPH) when SSRIs are used in the third trimester.^26^

Therefore, treatment with SSRIs in women of reproductive age should be accompanied by appropriate counselling and shared decision making as advised by national recommendations such as preconception discussions regarding contraception, risks of treatment during pregnancy and during breastfeeding, and possible discontinuation of SSRIs during pregnancy in cases of mild to moderate depression.^7,27,28^ However, rates of unplanned pregnancies have risen by 61% since the COVID-19 pandemic and are also more common in women living with depression; this challenges the provision of such preconception care and counselling.^29,30^ Thus, in the context of increasing SSRI use, there is a significant concern regarding potential unplanned SSRI exposure during pregnancy, and the associated rare but significant consequences highlighted by the MHRA.^24–26^

Previous work on other teratogenic medications regularly prescribed in primary care and feedback from women suggests such counselling is not often provided.^31,32^ Suggested possible reasons for this suboptimal care include lacks of time, opportunity, financial incentive, and prescriber knowledge.^33^

Primary care prescribers (including GPs and non-medical prescribers) issue most SSRIs in the UK and are guided by the *British National Formulary*, National Institute for Health and Care Excellence (NICE), royal colleges, and local prescribing formularies. Integrated Care Boards (ICBs) in England and Local Health Boards (LHBs) in Wales are responsible for generating and managing local prescribing formularies. Local formularies benefit from being able to update quickly in response to new safety concerns, acknowledge local population needs, improve cost-effective prescribing, and provide prescriber education; NICE recommends that regulator medicine safety advice be routinely included.^34,35^ Clinician adherence to local formulary guidance has been reported to be superior to other guidance sources, in view of the tailored contents local formularies can provide.^34^ Consequently, prescribing formularies are important in the landscape of prescribing resources available to primary care, and it is essential they reflect the risks of SSRI use around pregnancy outlined here.

We therefore sought to review local prescribing formularies across England and Wales with respect to prescribing of SSRIs in women of reproductive age, during pregnancy, and during breastfeeding.

We have used the term ‘women’ throughout; however, we acknowledge that our findings are relevant to all people who can become pregnant.

## Method

### Setting

Prescribing formularies generated and managed by ICBs (previously managed by clinical commissioning groups [CCGs]) in England and LHBs in Wales.

### Data collection

A list of CCGs in England and LHBs in Wales were identified using the NHS England and NHS Wales websites. A web search was then undertaken to identify individual CCG and LHB websites and their associated prescribing formularies in December 2021 (Supplementary data S1).

On 1 July 2022, all CCGs were abolished, and responsibility for providing NHS care on a local level, including prescribing formulary provision, was transferred to ICBs. Therefore, the above search strategy was repeated in July 2022 for ICBs and LHBs. All ICB websites and their associated formularies were reviewed (and LHB formularies were re-reviewed if any updates had occurred). Any formulary previously identified that was not also identified during our subsequent July 2022 review was removed. Only results from the July 2022 review are presented. Data are correct as of 9 December 2022.

Only documents or weblinks entitled ‘Formulary’ or ‘Prescribing Formulary’ were reviewed. If such documents or weblinks contained links or references to other documents, then these were also reviewed.

Excel spreadsheet data collection templates were piloted with a sample of formularies, and a codebook was developed. EL and AM-S each extracted data independently from 20% of formularies, and results were compared. Discrepancies were resolved by discussion between the data extractors, and with a third reviewer if required. A discrepancy rate of 3.51% was found; the majority being due to typographical or transcription error. The remaining 80% of formularies were reviewed by at least one reviewer.

For all formularies, the data outlined in Table 1 were extracted.

**Table 1.** Data points extracted for all formularies included in the review

Topic area	Detail of data extracted
ICB/LHB name	The name and location of the ICB/LHB (eg, NHS Dorset ICB)
Formulary location	The URL of the formulary was recorded and the method by which the formulary was located (via ICB/LHB website or keyword web search)
Date of extraction	The date the data were extracted from the formulary
Formulary version	The last date the formulary was updated
Formulary structure	How the formulary was structured; that is, by body system (eg, central nervous system), by medication class (eg, SSRI or antidepressant), or by individual medication name (eg, sertraline)

ICB = Integrated Care Board. LHB = Local Health Board. SSRI = selective serotonin reuptake inhibitor. URL = uniform resource locator.

If a formulary contained a listing for an SSRI with associated prescribing guidance for women of reproductive age, during pregnancy, or during breastfeeding (Supplementary data S2), then the following data were extracted:

- Source of guidance (for example, locally generated guidance or externally linked guidance to national bodies or organisations)
- Presence or absence of a hyperlink to, or description of, MHRA alerts regarding SSRI use in women of reproductive age, during pregnancy, or during breastfeeding

If a formulary contained an SSRI listing with associated locally generated guidance for women of reproductive age, during pregnancy, or during breastfeeding, then data were collected on recommended medication counselling and contraception, SSRI prescribing recommendations, risks of SSRI use, and advice regarding specialist services referrals (Table 2).

**Table 2.** Data points extracted from formularies containing locally generated guidance regarding SSRI prescribing in women of reproductive age, during pregnancy, or during breastfeeding

		Data points extracted for each patient group
Patient group	Women of reproductive age	Advice regarding first and second line SSRIs to be used in this group Advice regarding contraindicated SSRIs in this group Advice regarding counselling that HCPs should provide to this group regarding SSRIs Advice regarding contraception in this group in relation to SSRIs Advice regarding congenital abnormalities in this group in relation to SSRIs
	Pregnant women	Advice regarding first and second line SSRIs to be used in this group Advice regarding contraindicated SSRIs in this group Advice regarding counselling that HCPs should provide to this group regarding SSRIs
	Breastfeeding women	Advice regarding first and second line SSRIs to be used in this group Advice regarding contraindicated SSRIs in this group Advice regarding counselling that HCPs should provide to this group regarding SSRIs
	All (women of reproductive age AND pregnant women AND breastfeeding women)	Advice regarding contraception in relation to SSRIs Advice regarding congenital abnormalities in relation to SSRIs Advice regarding when to refer to specialist perinatal mental health Advice regarding risk PPHN with SSRI use Advice regarding neonatal withdrawal with SSRI use Advice regarding PPH risk with SSRI use

HCP = healthcare professional. PPH = postpartum haemorrhage. PPHN = persistent pulmonary hypertension of the newborn. SSRI = selective serotonin reuptake inhibitor.

### Data analysis

Data were collected and analysed using Excel (version 2208). Averages are presented as the mean and percentages rounded to one decimal place unless otherwise stated.

### Patient and public involvement

Patient and public involvement in preconception health research has previously been undertaken by EL and continued alongside this review. Patients and the public identified a need to explore the safety of teratogen prescribing in primary care, particularly regarding commonly prescribed medications such as SSRIs, and highlighted that improving our understanding of what guidance is available to prescribers is a key priority.

## Results

As of July 2022, 42 ICBs and seven LHBs were in existence in England and Wales. Thirty-nine of 42 ICBs and all LHBs either provided publicly accessible formularies on their website, or formularies were identified via keyword web search, or were made available following an email request. Three ICBs failed to respond to our request for formulary access. However, their previously associated CCG formulary remained active and updated; thus, data were extracted from these formularies in these instances.

**Figure 1.**
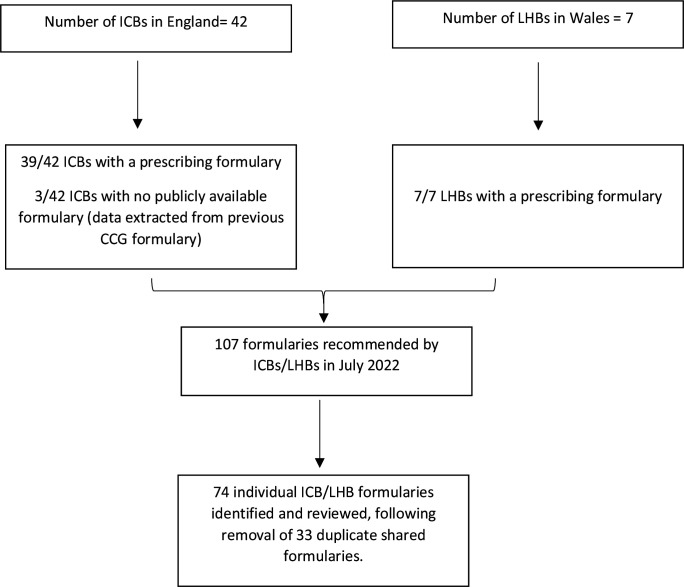
Flow chart of Integrated Care Boards in England and Local Health Boards in Wales and associated available prescribing formularies. CCG = clinical commissioning group. ICB = Integrated Care Board. LHB = Local Health Board.

A total of 107 formularies were recommended by ICBs/LHBs in July 2022; 33 were shared across different ICBs/LHBs. Following removal of duplicates, 74 individual ICB/LHB formularies were reviewed, and data were extracted (Figure 1). Of the 74 formularies reviewed, 25.7% (*n* = 19) displayed an update date. The oldest update date was 1 June 2012 and the most recent update was 1 November 2022.

### Provision of general SSRI prescribing guidance

Of those formularies that included SSRIs (*n* = 73), 93.2% (*n* = 68) contained some prescribing guidance, and 90.4% (*n* = 66) contained prescribing guidance for specific patient groups, such as older people or adolescents.

### Provision of SSRI prescribing guidance for women of reproductive age, during pregnancy, or during breastfeeding

**Figure 2.**
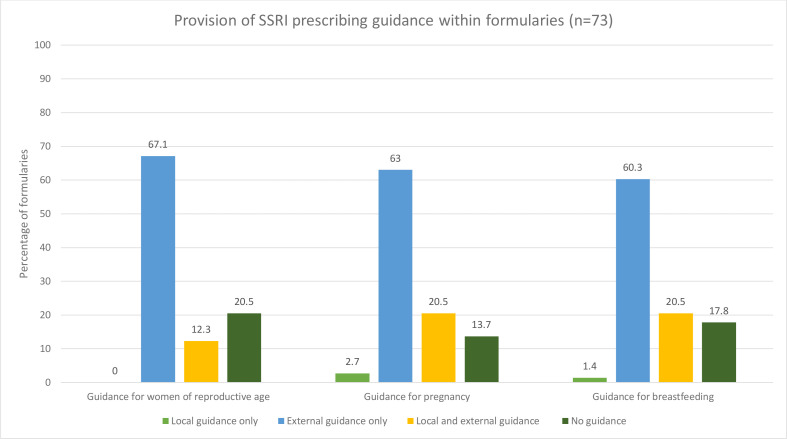
Percentage of formularies that provided locally generated guidance only, external guidance only, both locally generated and external guidance, or no guidance; for women of reproductive age, during pregnancy and during breastfeeding (*n* = 73 formularies). SSRI = selective serotonin reuptake inhibitor.

Of those formularies that contained SSRIs (*n* = 73), the majority contained some guidance for prescribing SSRIs in women of reproductive age (79.5%, *n* = 58), during pregnancy (86.3%, *n* = 63), or during breastfeeding (82.2%, *n* = 60). Figure 2 shows the percentage of formularies for each patient group that provided locally generated guidance, external guidance (most commonly via hyperlink to NICE guidance or MHRA guidance), or both.

Nine formularies provided local guidance for women of reproductive age; one recommended sertraline be prescribed first line and another stated paroxetine should not be used due to potential teratogenicity. Five formularies recommended that healthcare professionals (HCPs) should counsel women on contraception and two highlighted the potential future risk of congenital abnormalities with SSRI use.

Seventeen formularies provided local guidance for SSRI use in pregnancy, of which 58.8% (*n* = 10) and 41.2% (*n* = 7) provided advice on which SSRIs should be prescribed first-line and second-line, respectively: sertraline was recommended first line most commonly, followed by fluoxetine. A further 41.2% (*n* = 7) of these formularies advised against the use of fluoxetine during pregnancy. A minority of formularies (35.3%, *n* = 6) recommended that counselling should be provided to pregnant women when prescribing SSRIs.

Sixteen formularies provided local guidance for SSRI use during breastfeeding, of which 68.8% (*n* = 11) provided advice on which SSRIs should be prescribed first-line or second-line: sertraline was recommended first-line in all formularies, and citalopram and paroxetine were recommended equally frequently as second-line agents. Some formularies described some SSRIs as being contraindicated in breastfeeding, including citalopram, fluoxetine, and vortioxetine (18.8%, *n* = 3). A small number of these formularies (37.5%, *n* = 6) advised on what information HCPs should provide to women when prescribing SSRIs during breastfeeding.

In addition to the guidance outlined for specific patient groups above, a further eight (11.0%) formularies highlighted the risk of congenital abnormalities with SSRI use, nine (12.3%) provided advice regarding the risk of neonatal serotonergic effects or withdrawal, and eight (11.0%) advised on referral criteria for specialist services. However, it was unclear whether these guidance items were intended for women of reproductive age, during pregnancy, or during breastfeeding.

### MHRA alerts

**Figure 3.**
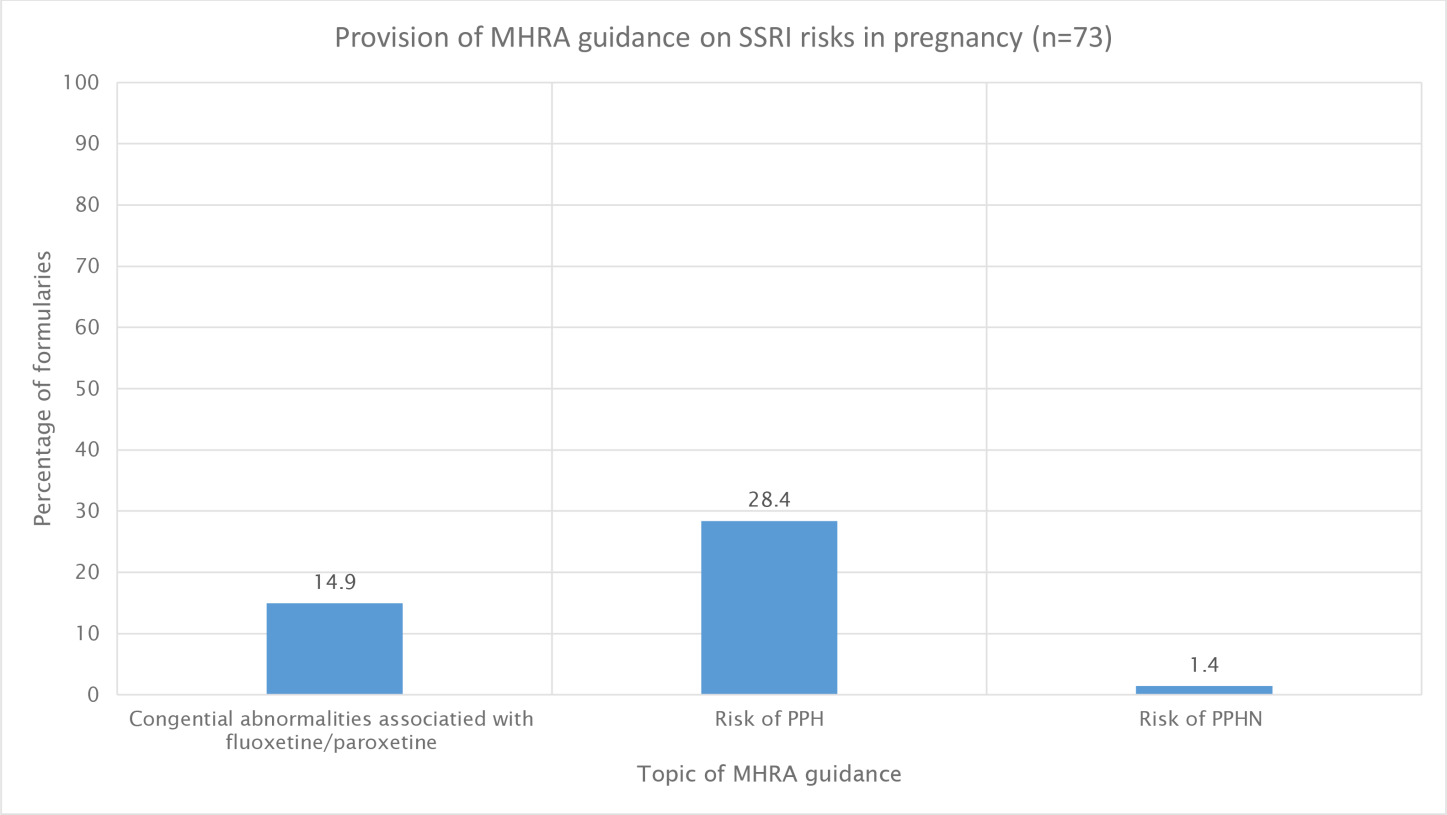
Percentage of formularies that included a hyperlink to, or a description of the contents of, specific MHRA alerts regarding SSRI use (*n* = 73 formularies). MHRA = Medicines and Healthcare products Regulatory Agency. PPH = postpartum haemorrhage. PPHN = persistent pulmonary hypertension in the newborn. SSRI = selective serotonin reuptake inhibitor.

Formularies containing SSRI prescribing guidance were reviewed for the inclusion of a hyperlink to, or a description of the contents of, specific MHRA alerts regarding SSRI use, including the risk of congenital abnormalities, PPH, and PPHN (Figure 3).

## Discussion

### Summary

The majority of formularies reviewed provided some SSRI prescribing guidance for women of reproductive age (79.5%, *n* = 58), during pregnancy (86.3%, *n* = 63), or during breastfeeding (82.2%, *n* = 60). However, this was largely via hyperlinks to external sources, which may be easily missed or overlooked by clinicians. In those formularies where local guidance was provided, only just over half of formularies recommended prescribers undertake discussions regarding contraception in women of reproductive age, and just over one-third advised prescribers to counsel women regarding SSRI use during pregnancy. Furthermore, of the formularies that provided local guidance, 11.1% recommended specific first-line SSRIs for women of reproductive age, 58.8% for women during pregnancy, and 68.8% for women during breastfeeding; and the medication recommended varied considerably. This contrasts with national advice, which does not make similarly specific recommendations. Such discordance is likely to cause confusion to prescribers and is concerning if formularies are relied on solely for medication safety information.

Concerningly, our review also identified poor translation and communication of MHRA alerts regarding SSRI use into prescribing formularies; only 14.9% of formularies included or referred to the MHRA alert regarding congenital abnormalities, 28.4% the risk of PPH, and 1.4% the risk of PPHN.^35^

### Strengths and limitations

Our review is the first UK-based study to reveal the large gaps in provision of prescribing advice within local formularies regarding SSRI use in women of childbearing age, during pregnancy, and during breastfeeding. Our systematic approach and low inter-reviewer discrepancy rate supports our important findings to be accurate and subject to minimal interpretation error.

Due to the continuously changing landscape of local healthcare provision and organisation, including provision of prescribing formularies and clinical updates to such formularies, it is challenging to present a contemporaneous national picture across 49 ICBs/LHBs hosting 74 formularies between them, at any one time. Therefore, in the time elapsed between data collection and publication, formularies may have been updated. However, our repeat review of all formularies in July 2022, following the CCG-to-ICB transition period, revealed no changes regarding SSRI prescribing guidance. Our review was limited to formularies in England and Wales, which may limit the international generalisability of our results. Furthermore, only prescribing formulary websites and guidance documents explicitly linked to these websites (that is, by functioning hyperlink) were included in our review; prescribing guidance may be available elsewhere.

### Comparison with existing literature

Previous studies have found that women are keen to discuss medication use in relation to pregnancy with their prescribers; however, teratogenic medication counselling is rarely given in primary care nor recorded.^31,32^ This is the first UK-based review to provide some insight into why prescribers may not be providing such information to their patients: possibly due to suboptimal and contradictory sources of local formulary prescribing advice. Thus, this review provides essential groundwork for further quantitative and qualitative work (already underway by the authors), to better understand the facilitators and barriers to providing such medication counselling in primary care and allow for future intervention development.

Our finding of contradictory prescribing guidance between local and national sources is congruent with results from other systematic reviews of formulary guidance on different clinical topics.^36,37^ This may be the result of a large number of CCGs merging and then transitioning to ICBs within the past 5 years, resulting in amalgamation of various local sources of information.

### Implications for research and practice

Our results provide two main considerations for future research, clinical practice and future policy. First, we and others have demonstrated that locally produced prescribing guidance, if it is available, is often outdated and contradictory to that produced by national bodies. Our results draw into question the utility of prescribing formularies in providing medicines advice, and highlight their potential for causing confusion among prescribers; thus, potentially contributing to suboptimal clinical management. We conclude their position as a guidance provider should be reconsidered in future policy reviews of local healthcare provision.

Second, we acknowledge the critical importance of pharmacologically treating maternal mental illness. However, accompanying adequate medication counselling is essential. Our results not only suggest suboptimal provision of prescribing advice for SSRIs, increasing the risk of inadvertent pregnancy exposure, but also highlight a wider issue of inadequate provision for preconception care and teratogen counselling within primary care. In the context of increasing SSRI prescription rates, along with rates of unplanned pregnancy, our results have significant implications for current practice and policy. Studies to further elucidate the risks of a variety of teratogenic medications are underway.^38^ Further research to inform policy, involving patients and prescribers to ascertain how preconception care can be provided in an already overburdened healthcare system, is required to improve maternal and child outcomes.

## Data

The dataset relied on in this article is available from the corresponding author on reasonable request.

## References

[1] Cleary BJ, Butt H, Strawbridge JD (2010). Medication use in early pregnancy-prevalence and determinants of use in a prospective cohort of women. Pharmacoepidemiol Drug Saf.

[2] Wise J (2014). One in 10 women in England takes antidepressants, survey shows. BMJ.

[3] NHS Digital (2015). Health Survey for England, 2014.

[4] Anderson KN, Lind JN, Simeone RM (2020). Maternal use of specific antidepressant medications during early pregnancy and the risk of selected birth defects. JAMA Psychiatry.

[5] Lee SI, Azcoaga-Lorenzo A, Agrawal U (2022). Epidemiology of pre-existing Multimorbidity in pregnant women in the UK in 2018: a population-based cross-sectional study. BMC Pregnancy Childbirth.

[6] Subramanian A, Azcoaga-Lorenzo A, Anand A (2023). Polypharmacy during pregnancy and associated risk factors: a retrospective analysis of 577 medication exposures among 1.5 million pregnancies in the UK, 2000-2019. BMC Med.

[7] National Institute for Health and Care Excellence (2023). Depression in adults: treatment and management. NICE guideline [NG222].

[8] National Institute for Health and Care Excellence (2011). Generalised anxiety disorder and panic disorder in adults: management.

[9] MacKenna B (2019). What are the most commonly prescribed medicines? Top 10 prescribed medicines in NHS England primary care for 2019.

[10] Heald AH, Stedman M, Davies M (2020). Antidepressant prescribing in England: patterns and costs. Prim Care Companion CNS Disord.

[11] Iacobucci G (2019). NHS prescribed record number of antidepressants last year. BMJ.

[12] Public Health England (2020). Prescribed medicines review: summary.

[13] Lalji HM, McGrogan A, Bailey SJ (2021). An analysis of antidepressant prescribing trends in England 2015-2019. J Affect Disord Rep.

[14] Bogowicz P, Curtis HJ, Walker AJ (2021). Trends and variation in antidepressant prescribing in English primary care: a retrospective longitudinal study. BJGP Open.

[15] Archer C, MacNeill SJ, Mars B (2022). Rise in prescribing for anxiety in UK primary care between 2003 and 2018: a population-based cohort study using Clinical Practice Research Datalink. Br J Gen Pract.

[16] NHS Business Services Authority (2023). Medicines Used in Mental Health – England – 2015/16 to 2021/22.

[17] Heald AH, Stedman M, Davies M (2020). Influences on the use of antidepressants in primary care: all England general practice-level analysis of demographic, practice-level and prescriber factors. Hum Psychopharmacol.

[18] Chan J, Natekar A, Einarson A, Koren G (2014). Risks of untreated depression in pregnancy. Can Fam Physician.

[19] Dalfen AK, Goldbloom DS, Davine J (2019). Psychiatry in Primary Care A Concise Canadian Pocket Guide (2nd edition).

[20] Jarde A, Morais M, Kingston D (2016). Neonatal outcomes in women with untreated Antenatal depression compared with women without depression: a systematic review and meta-analysis. JAMA Psychiatry.

[21] Su J-A, Chang C-C, Yang Y-H (2023). Neonatal and pregnancy complications following maternal depression or antidepressant exposure: a population-based, retrospective birth cohort study. Asian J Psychiatr.

[22] Knight MBK, Felker A, Patel R (2021). Saving lives, improving mothers’ care core report - lessons learned to inform maternity care from the UK and Ireland confidential enquiries into maternal deaths and morbidity 2019-21.

[23] Knight MBK, Patel R, Shakespeare J (2022). Saving lives, improving mothers’ care core report - lessons learned to inform maternity care from the UK and Ireland confidential enquiries into maternal deaths and morbidity 2018-20.

[24] UK Government (2023). Fluoxetine: possible small risk of congenital cardiac defects.

[25] UK Government (2023). SSRIs and SNRIs: risk of persistent pulmonary hypertension in the newborn.

[26] UK Government (2023). SSRI/SNRI antidepressant medicines: small increased risk of postpartum haemorrhage when used in the month before delivery.

[27] National Institute for Health and Care Excellence (2023). Antenatal and postnatal mental health: clinical management and service guidance.

[28] Royal College of Obstetricians and Gynaecologists (2023). Management of women with mental health issues during pregnancy and the postnatal period (Good Practice No.14).

[29] Balachandren N, Barrett G, Stephenson JM (2022). Impact of the SARS-Cov-2 pandemic on access to contraception and pregnancy intentions: a national prospective cohort study of the UK population. BMJ Sex Reprod Health.

[30] Wellings K, Jones KG, Mercer CH (2013). The prevalence of unplanned pregnancy and associated factors in Britain: findings from the third national survey of sexual attitudes and lifestyles (Natsal-3). Lancet.

[31] Lovegrove E, Robson J, McGettigan P (2020). Pregnancy protection and pregnancies in women prescribed ACE inhibitors or ARBs: a cross-sectional study in primary care. Br J Gen Pract.

[32] Sanders J, Blaylock R, Dean C (2023). Women’s experiences of over-the-counter and prescription medication during pregnancy in the UK: findings from survey free-text responses and narrative interviews. BMJ Open.

[33] Schwarz EB, Santucci A, Borrero S (2009). Perspectives of primary care clinicians on teratogenic risk counseling. Birth Defects Res A Clin Mol Teratol.

[34] Reynolds DJM, Fajemisin O, Wilds S (2012). Local Formularies. Br J Clin Pharmacol.

[35] National Institute for Health and Care Excellence (2014). Developing and updating local formularies medicines practice guideline [MPG1].

[36] Amakye NYT, Chan J, Ridd MJ (2022). Emollient prescribing Formularies and guidelines in England, 2021: a cross-sectional study. Clin Exp Dermatol.

[37] Chan JP, Boyd G, Quinn PA, Ridd MJ (2018). Emollient prescribing formularies in England and Wales: a cross-sectional study. BMJ Open.

[38] ConcePTION (2023). The Work Packages.

